# Cytokine detection and simultaneous assessment of rheumatoid factor interference in human serum and synovial fluid using high-sensitivity protein arrays on plasmonic gold chips

**DOI:** 10.1186/s12896-015-0186-0

**Published:** 2015-08-13

**Authors:** Manfè Valentina, Fleckner Jan, Nørby Lisby Peder, Zhang Bo, Dai Hongjie, Keller Pernille

**Affiliations:** Department of PharmacoGenetics, Biopharmaceutical Research Unit, Novo Nordisk A/S, Novo Nordisk Park 1, Maaloev, 2760 Denmark; Department of Chemistry, Stanford University, San Francisco, CA USA

**Keywords:** Protein microarray, Cytokines, IL-20, Rheumatoid arthritis, Osteoarthritis, Rheumatoid factor interference, Plasmonic gold, Fluorescence enhancement

## Abstract

**Background:**

Fluorescence-enhancing microarray on plasmonic gold film is an attractive alternative to traditional enzyme-linked immunosorbent assay (ELISA) for cytokine detection because of the increased sensitivity. The assay chemistry is similar to an ELISA sandwich assay, but owing to the gold substrate, cytokine measurements are 10 to 100 times more sensitive and can be multiplexed. Plasmonic protein microarrays are, as other immunoassays, affected by the presence of heterophilic antibodies and rheumatoid factor may lead to analytical errors with serious implications for patient care. Here, we present a plasmonic gold substrate protein microarray for high-sensitivity detection of cytokines with simultaneous assessment of rheumatoid factor interference on a single chip.

**Results:**

Paired serum and synovial fluid samples from patients with rheumatoid arthritis (*n* = 18), osteoarthritis (*n* = 9) or healthy controls (*n* = 10) were arrayed on near-infrared fluorescence enhancing plasmonic gold chips spotted with cytokine-specific capture antibody and isotype control antibody. Possible rheumatoid factor interference was visualised by a non-specific signal from the isotype control antibody, and pre-treatment of samples with heat-aggregated animal IgG eliminated this background contamination. The platform was optimised using the cytokine IL-20. The protein microarray platform allowed for the detection of human IL-20 at levels <1 pg/ml with reliable IL-20 quantification over a 5-log dynamic range. Samples for which rheumatoid factor caused artefacts were identified and a method for eliminating rheumatoid factor interference was developed and validated. IL-20 protein levels were significantly higher in synovial fluid samples from patients with rheumatoid arthritis compared to osteoarthritis (*p* < 0.001), while serum levels of IL-20 did not differ between patients with rheumatoid arthritis, osteoarthritis or healthy controls.

**Conclusion:**

Using novel plasmonic gold chips, we developed a highly sensitive and accurate assay platform to detect lowly expressed cytokines in biological fluids, allowing for the elimination of rheumatoid factor interference in as little as 5 μl sample volume. The detection limit was below 1 pg/ml for IL-20 and linearity was achieved over a 5-log dynamic range. This technology is highly advantageous for cytokines where sensitivity or sample volume is critical or where assessment of rheumatoid factor interference needs addressed and eliminated.

## Background

Cytokines are low molecular weight proteins that exert pleiotropic effects on cells involved in the immune and acute phase responses. Because cytokines are crucially involved in many biological processes such as hematopoiesis, immunity, tumour genesis, haemostasis, vascularization and repair of connective tissues, deregulation of cytokines is implicated in the pathophysiology of a variety of human diseases and may be used to refine diagnosis, measure the progress of diseases, or predict and monitor the effects of treatment [1]. Cytokine measurements using enzyme-linked immunosorbent assay (ELISA), the current standard procedure for protein quantification [2], is often limited by the requirement of high sample volume (at least 50 μL), narrow dynamic range and low throughput due to the analysis of a single analyte per sample [3]. Compared with ELISA, protein microarray technology on fluorescence-enhancing plasmonic gold films recently described by Tabakman et al. [4] provides improved sensitivity and a broader dynamic range while allowing for high-throughput analysis of multiple determinants within a single sample [5–7].

We wanted to optimise this platform to work on cytokines that are lowly expressed. We further wanted to apply the technology on biological fluids from patients with rheumatoid arthritis (RA) where samples are often scarce in volume and may be compromised by the presence of heterophilic antibodies. We chose IL-20 as a model cytokine due its widely described role in the pathogenesis of several autoimmune diseases, including psoriasis and RA [8]. IL-20 is up-regulated in psoriatic skin [9], whereas in RA patients, it is expressed in synovial fibroblasts, mononuclear cells and neutrophil granulocytes of the synovial membrane [10]. No evidence supports a role for IL-20 in the systemic immune response, suggesting that IL-20 acts at local sites of inflammation in RA patients. Finally, IL-20 is involved in tumour growth and metastasis and represents a promising target in the treatment of breast, bladder and lung cancers [11–13].

IL-20 protein levels in human biological fluids reportedly range from 60 pg/ml to 1650 ng/ml [10, 14, 15]. For ELISA measurements, multiple sample dilutions are required to cover this range, making reliable measurements of serum/plasma and synovial fluid (SF) IL-20 challenging. Heterophilic antibodies, including Rheumatoid Factor (RF), further compromise data reliability, as RF causes falsely increased or false positive results when the cytokine is detected using traditional immunoassays, *e.g.* ELISA, or bead based assays [16]. RF mainly interferes with the measurement of cytokines by cross-linking capture and detection antibodies [17]. RF has a prevalence of up to 80 % in RA patients and 15 % in healthy individuals [17, 18], thus there is a tremendous need for novel sensitive assays that can assess and eliminate potential RF interference while having a broad detection range.

Here, we show that by exploiting the near-infrared physical fluorescence enhancement provided by a gold film, plasmonic protein microarrays can detect cytokines at sensitivities <1 pg/ml while covering a 5-log dynamic range. On the same chip, RF interference was assessed and false positive results caused by RF were eliminated. The sample volume required was low (<5 μl), providing major advantages when limited amounts of biological materials are available.

## Results

### Detection of IL-20 protein levels using plasmonic gold substrates

**Fig 1:**
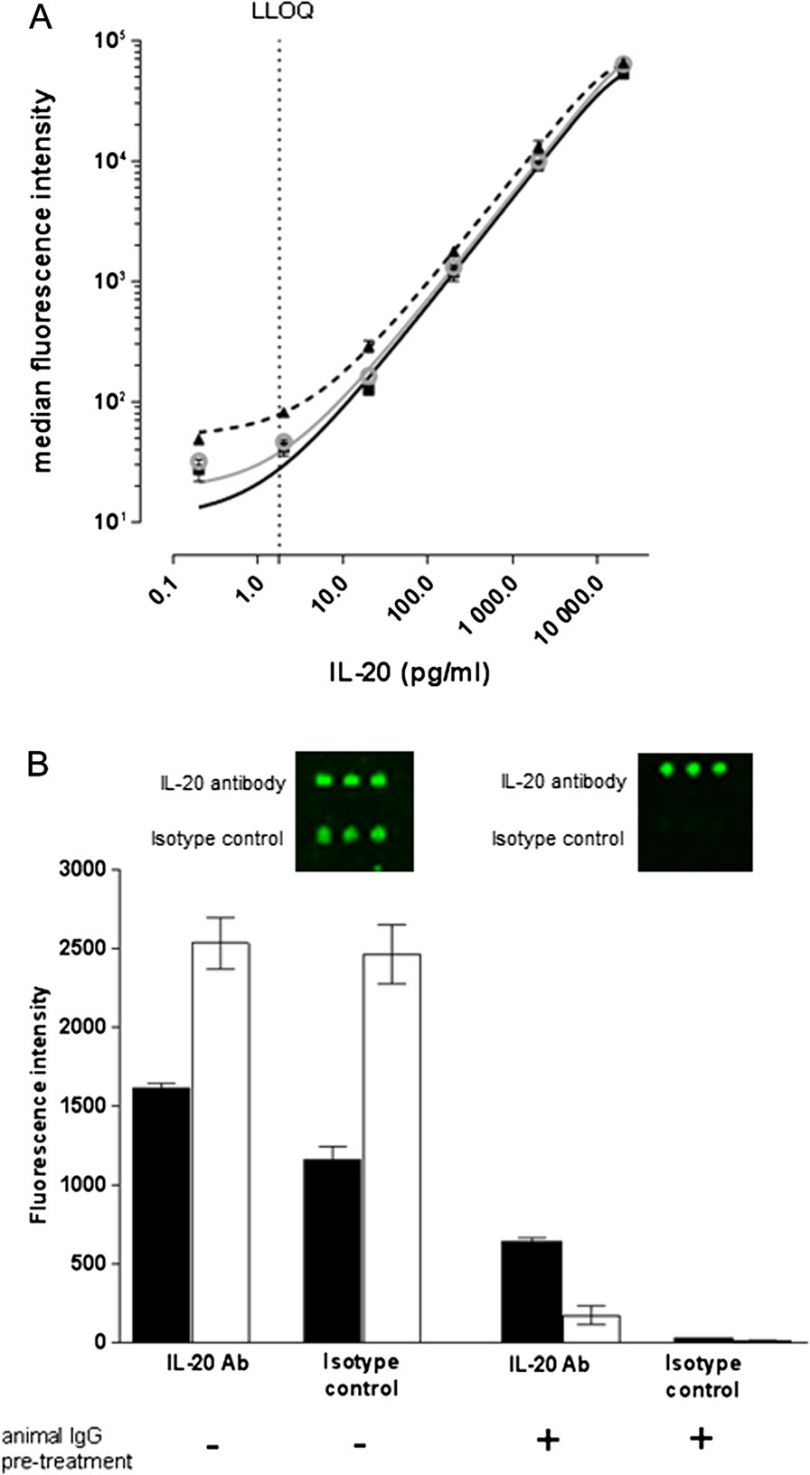
IL-20 quantification using plasmonic gold substrates. **a**) IL-20 standard curves obtained using rh-IL-20 expressed in *E.Coli* (produced by Novo Nordisk A/S (), or R&D Systems ()) or in HEK-293 (Novo Nordisk A/S ()). rh-IL-20 was diluted in 5 % BSA in PBS. Median ± SD fluorescence intensities are shown. Lower limit of quantification (LLOQ) is indicated by the vertical dotted line. **b**) Fluorescence intensities for IL-20 and isotype control capture antibodies before and after pre-incubation with a mix of animal IgGs. Serum (black) and synovial fluid (white) mean IL-20 levels ± SD for two donors are shown. Representative maps (inserts) shown for one donor

Highly similar standard curves were obtained from recombinant human (rh)-IL-20 expressed in *E.Coli* (produced by Novo Nordisk A/S or by R&D Systems) or HEK-293 (produced by Novo Nordisk A/S) (Fig. 1a), suggesting that post-translational modifications do not interfere with detection of IL-20. Thus, IL-20 levels were determined based on *E.Coli*-expressed rh-IL-20 (Novo Nordisk A/S). Levels of IL-20 in the subject samples ranged from 11–650 pg/ml.

Detection of IL-20 showed linearity over a 5-log dynamic range covering 2–20,000 pg/ml (r^2^ > 0.99) (Fig. 1a), allowing for accurate cytokine quantification without the need for multiple dilutions. Limit of detection (LOD) was 0.2 pg/ml. Lower limit of quantification (LLOQ) was 2 pg/ml, while the accuracy of the back-calculated calibrator concentration was 91 % (87-95 %) and precision (coefficient of variation, CV%) was <20 %. Similar sensitivities and dynamic ranges were obtained for other cytokines; IL-6, TNFα, IL-1β and IL-10 (data not shown). Spike-in recovery measurements were performed in serum samples from three healthy controls (HC) and were 93 % (72-113 %), which is within the technical specification range of 70-130 % [2].

### Elimination of RF interference

Spike-in recovery of cytokines in serum samples from RA patients were affected by the presence of RF (data not shown), highlighting the need for an estimate of RF interference and a protocol for eliminating the erroneous signal amplification. Regardless of whether anti-IL-20 or isotype control was used as capture antibody, the fluorescence signals were similar for two RA serum samples (Fig. 1b), indicating that RF interfered with IL-20 detection resulting in false positive signals. Pre-treatment of samples with a mix of animal IgGs that were previously heat aggregated, eliminated the RF interference (Fig. 1b).

### Levels of IL-20 in serum and synovial fluid of RA patients

**Fig 2:**
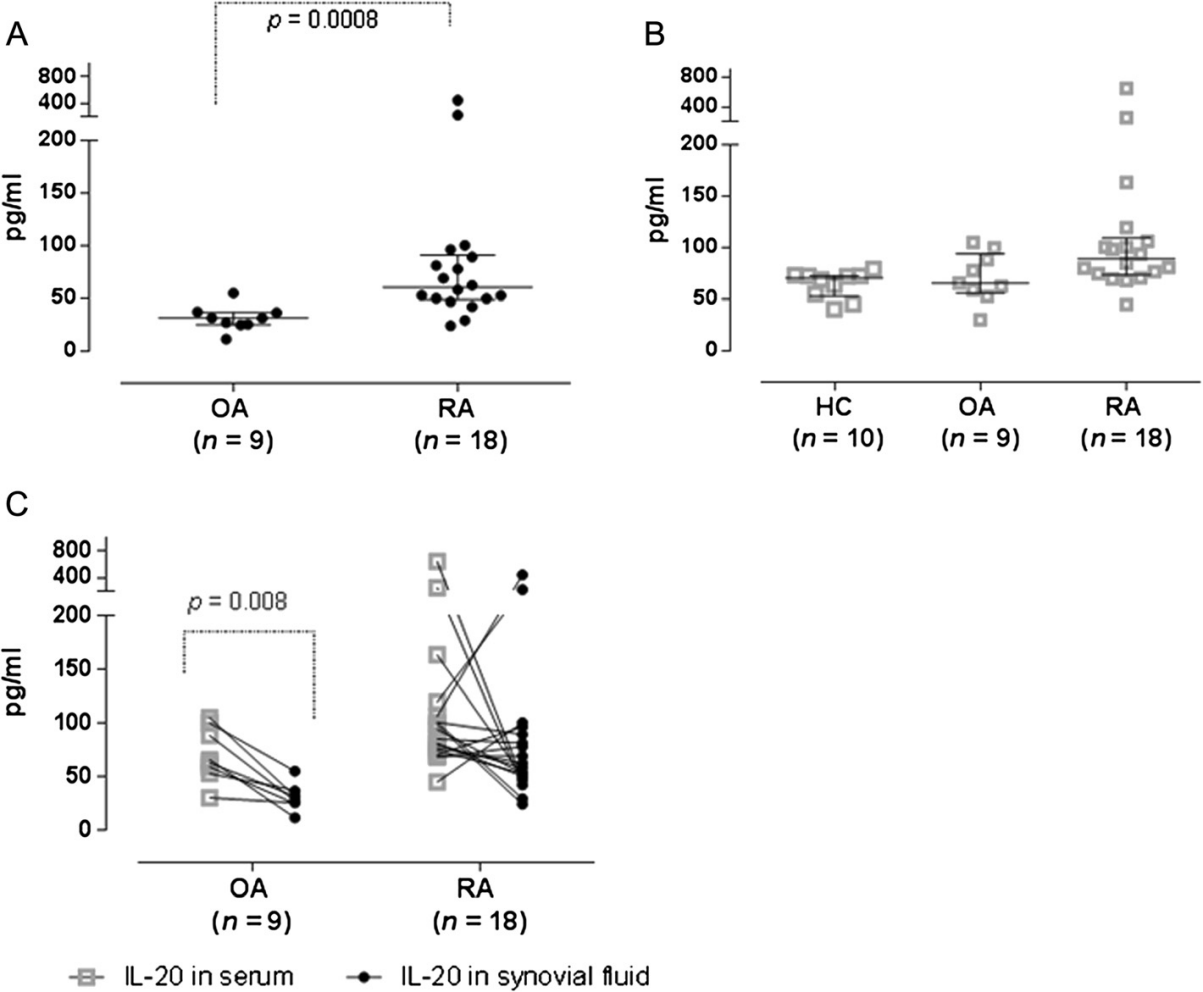
IL-20 levels in synovial fluid and serum samples. **a**) IL-20 levels are higher in synovial fluid samples from patients with RA compared with OA (*p* < 0.001). Each dot represents the average concentration for 1 patient based on 3 independent experiments, median and interquartile range is shown. **b**) IL-20 levels in serum samples from RA, OA and HC. Data are represented as in (**a**). **c**) IL-20 levels in matched serum and synovial fluid samples from OA and RA patients. Data are represented as in (**a**). IL-20 serum levels in OA patients were higher than matched SF levels (*p* = 0.008)

IL-20 levels in SF were significantly higher (*p* < 0.001) in RA patients (60 pg/ml, 24–445 pg/ml) than in patients with osteoarthritis (OA) (32 pg/ml, 11–55 pg/ml) (Fig. 2a). However, IL-20 levels in serum were comparable for RA (89 pg/ml, 45–643 pg/ml), OA (66 pg/ml, 30–105 pg/ml), or HC (71 pg/ml, 40–80 pg/ml) (Fig. 2b). Pairwise comparison showed that the serum levels of IL-20 were higher than the corresponding SF levels in OA patients (*p* = 0.0078), while this was not the case in RA patients (*p* = 0.167; Fig. 2c).

## Discussion

RF interference in immuno-assays is a significant source of uncertainty in medical testing and may cause misleading interpretation of the results for RA patients [19]. So far, detection of RF interference has required measurement of the same sample before and after addition of a blocking agent [20] or following sample pre-treatment in non-immune serum [21]. However these tests require a large sample volume (up to 400 μl) [19], and may be inconclusive or require re-collection of sample material. This study provides a novel protein microarray platform based on plasmonic gold films that reliably detects cytokines and assess for RF interference in 5 μl sample volume.

This study focused on IL-20 due to its pro-inflammatory role in autoimmune diseases and because this cytokine requires a high-sensitivity assay for detection in SF where limited sample material is available. However, the technology can be applied to other cytokines provided that good capture and detection antibody pairs can be obtained. We have further optimised the assay for IL-6, TNFα, IL-1β and IL-10.

IL-20 levels in SF have been measured using ELISA [10, 22]. However data reliability needs to be confirmed by negating an effect of RF [16], as traditional immuno-assays produce high background noise if RF is present [4]. By analysing serum and SF samples in arrays spotted both with anti-IL-20 antibody and isotype control antibody, we could assess for false positive measurements and show the successful elimination of RF interference. The use of plasmonic gold substrates provided fluorescence enhancement, lowering the LOD to 0.2 pg/ml, a level ~100 times lower than a traditional ELISA kit (Human IL-20 Quantikine ELISA Kit, R&D systems, LOD is ~20 pg/ml). Plasmonic gold chips also supported a wider dynamic range covering linear IL-20 detection over a 5-log dynamic range (2–2000 pg/ml, r^2^ > 0.99) and inter and intra-runs CV of <20 %.

Similarly to data by Hsu et al. [10], we showed that IL-20 levels were significantly higher in SF samples from patients with RA compared to OA (*p* < 0.001). Our findings were in contrast with those of Scrivo et al. [22], who reported increased IL-20 levels in serum of RA compared to OA and HC. Of note, the subjects included in the latter study are not comparable to ours in terms of number and clinical features. Moreover, RF interference was not assessed in the paper by Scrivo et al. The IL-20 increase in SF of RA patients rather than in the circulation supported a role for IL-20 at the site of inflammation rather than in the systemic immune response.

Although traditional ELISA remains the method of choice for cytokine detection in clinical laboratories, the presence of RF will lead to analytical errors with serious implications for patient care [23]. We show that artefacts caused by RF assay interference can be eliminated by a simple sample pre-treatment using heat-aggregated animal IgG. RF elimination can be done without affecting assay sensitivity or cytokine detection. This platform was validated using serum and SF samples from patients with RA, OA and HC. Our protein microarrays on plasmonic gold substrates can be deployed to assess and eliminate RF interference to finally obtain reliable cytokine data for use in the clinic and in research to increase quality of patient care.

## Conclusions

This study showed that plasmonic protein microarrays on gold film substrate allowed for sensitive and reliable cytokine quantification in biological fluids in 5 μl sample volume. On the same chip, rheumatoid factor interference can be assessed and eliminated without requirement of extra sample material or additional immunoassays. Hence, this technology can be applied to reliably detect lowly expressed cytokines in different biological sources even if limited amounts of biological materials are available.

## Methods

### Clinical characteristics of patient groups

Samples from HC (*n* = 10, 5 female, 5 men) were from BioreclamationIVT (West Sussex, UK), while samples from patients with RA (*n* = 18, 12 female, 6 men) and OA (*n* = 9, 6 female, 3 men) were from DVBiologics (Costa Mesa, CA, USA). Mean age (±SD) for both RA and OA groups was 65 (±13). Of the 18 RA patients, 17 were RF positive (mean titer ± SD; 26 ± 12). All RA patients were CRP positive (mean ± SD; 11 ± 5 mg/dl). Disease Activity Score in 28 Joints was >2.6 for all RA patients indicating no remission cases. Validation of the RF removal protocol was performed on 3 additional RA samples (DVBiologics).

Written informed consent was obtained for all subjects according to the Declaration of Helsinki and the General Assembly Revision of 2008. The study has been approved by the Ethics Committee of Copenhagen and Frederiksberg.

### Protein array reagents

The following reagents were from Sigma–Aldrich (St. Louis, MO, USA); 16-mercaptohexadecanoic acid, cysteamine, 1-ethyl-3-(3-dimethylaminopropyl) carbodiimide, succinic anhydride and N-hydroxysuccinimide. Triethylamine, dimethylformamide and bovine serum albumin (BSA) were from Fisher Scientific (Pittsburgh, PA, USA). Six-armed-poly(ethyleneglycol)–amine was from SunBio (South Korea). PBS (Cat#70011-036) and Tween-20 were from Life Technologies (Grand Island, NY, USA).

Sandwich antibody pairs for IL-20 were produced by Novo Nordisk A/S. Capture anti-IL-20 antibody was of the IgG1 isotype. Unspecific binding was assessed using a IgG1 isotype control from R&D Systems (MAB002, Minneapolis, MN, USA). IRDye-800 streptavidin conjugate was from LiCor Biosciences (Lincoln, NE, USA). Mouse, goat and bovine IgG were from Jackson ImmunoResearch (West Grove, PA, USA).

Purified cytokine antigen standards and sandwich antibody pairs for IL-1β (MAB601, BAF201), IL-10 (MAB2172, BAF217), IL-6 (MAB206, BAF206), and TNFα (MAB610, BAF210) were purchased from R&D systems. All capture antibodies were of the IgG1 isotype.

### rh-IL-20 protein standards

Rh-IL-20 derived from *E.Coli* cells was purchased from R&D systems (Cat#1102-IL-025) or produced at Novo Nordisk A/S. Rh-IL-20 produced in HEK-293 cells was from Novo Nordisk A/S. The activity of *E.coli* expressed Met-IL-20 and HEK-293 expressed IL-20 was determined by a IL-20R1/IL-20R2 Reporter Gene Assay (STAT-Luc) showing EC_50_ of 896 and 819 pg/ml, respectively (data not shown). The protein standards were diluted from 1 nM to 10 fM (17600–0.176 pg/ml) using 10-fold dilution steps in 5 % BSA in PBS.

### IL-20 assay

Chemically modified plasmonic gold slides were prepared as described [5]. Capture antibody or isotype control antibody (3 μM in PBS + 1 % glycerol) were printed in triplicate spots using Bio-Rad VersArray Chipwriter and solid pins (Arrayit, USA) at 25 °C and 60 % humidity, resulting in microarray feature diameters of ~400 μm. Slides were blocked overnight at 4 °C in 5 % BSA in PBS. Samples (5 μl) were diluted 4-fold in PBS and applied to each micro-well and incubated for 4 h at RT. Slides were washed twice in 0.5 % Tween-20 in PBS, once with PBS, and 5 nM human biotinylated polyclonal antibody in 10 % BSA in PBS was incubated over each micro-well for 1 h at RT. Slides were washed as before and 2 nM IR800 conjugated streptavidin in 10 % BSA in PBS solution added to the slides for 1 h at RT. After another wash, slides were rapidly immersed in deionized water and dried with compressed air.

### Fluorescence measurement and data analysis

Slides were scanned using Odyssey scanner (Li-cor Biosciences) as described [5]. Fluorescence intensities were background-subtracted using the global background subtraction method (GenePixPro V6), before calculating the median pixel intensities for features printed in triplicates. Standard curves were obtained using 5-parameter logistic curve. Limit of detection (LOD) was defined as the concentration at three standard deviations above the mean fluorescence of the blank. Lower limit of quantification (LLOQ) was defined as the lowest concentration at which the recovery of each calibrator was within 70-130 % and the inter-run coefficients of variation (CV) was <20 % [24]. The assay range was defined as the concentration range in which the cytokine measurements were linear (r^2^ > 0.99), inter and intra-run CV was 20 %, respectively, and spike recoveries between 70-130 %. All cytokine measurements are reported as median and range.

Statistical analysis was performed using GraphPad Prism V.6 by applying a Mann–Whitney test. Wilcoxon matched-pairs test was used to compare IL-20 levels in matched serum and synovial fluid of each subject. *P*-values <0.05 were considered statistically significant.

### Spike recoveries

Serum samples from three of the HCs were spiked with 20 or 2000 pg/ml of rh-IL-20 (produced in *E.Coli* by Novo Nordisk A/S). Spike recoveries were obtained by subtracting the observed concentration of the un-spiked sample (observed un-spiked) from the observed concentration of the added sample (observed spiked), and divided by the known concentration of the spiked sample (expected spiked).

### Removal of RF interference

By using an isotype control capture antibody, non-specific signal due to RF can be identified [20, 21]. To remove RF from biological samples showing RF-interference, we applied a modified protocol of Kragstrup et al. [20], where samples were diluted in an assay diluent containing a mix of animal IgG (mouse, bovine and goat, each at 50 μg/ml in PBS) previously heat-aggregated at 60 °C for 30 min. Serum samples were diluted 4-fold in the assay diluent containing animal IgG and incubated 30 min at RT. Samples were added to the gold slides and IL-20 detection performed as described above. Cytokine measurements were unaffected by addition of animal IgGs to serum samples, as spike-in recoveries for samples with and without animal IgG-treatment (*n* = 3, RA donors) were within the accepted range of 70–130 % (IL-6, 99 % (94–103 %); IL-1β 93 % (92–95 %); TNFα, 98 % (84–112 %) and IL-10, 88 % (86–89 %).

For the analysis of RA, OA and HC samples, RF interference assessment was performed and interference identified for two RA samples for which the RF elimination protocol was applied.

## Abbreviations

ELISA: Enzyme-linked immunosorbent assay
RA: Rheumatoid arthritis
SF: Synovial fluid
RF: Rheumatoid factor
rh: Recombinant human
LOD: Limit of detection
LLOQ: Lower limit of quantification
CV: Coefficient of variation
HC: Healthy control
OA: Osteoarthritis
BSA: Bovine serum albumin
